# Wavelet-Based Fractal Analysis of rs-fMRI for Classification of Alzheimer’s Disease

**DOI:** 10.3390/s22093102

**Published:** 2022-04-19

**Authors:** Alishba Sadiq, Norashikin Yahya, Tong Boon Tang, Hilwati Hashim, Imran Naseem

**Affiliations:** 1 Centre for Intelligent Signal and Imaging Research (CISIR), Electrical and Electronic Engineering Department, Universiti Teknologi PETRONAS, Seri Iskandar 32610, Malaysia; alishba_18001778@utp.edu.my (A.S.); tongboon.tang@utp.edu.my (T.B.T.); 2 Radiology Department, Hospital UiTM, Sungai Buloh 47000, Malaysia; hilwa167@uitm.edu.my; 3 College of Engineering, Karachi Institute of Economics and Technology, Karachi 75190, Pakistan; imrannaseem@pafkiet.edu.pk; 4 Department of Electrical, Electronic and Computer Engineering, The University of Western Australia, 35 Stirling Highway, Crawley, WA 6009, Australia; 5 Research and Development, Love for Data, Karachi 75270, Pakistan

**Keywords:** fractal connectivity, fractal integrated process, Hurst exponent, nonfractal connectivity, Pearson correlation

## Abstract

The resting-state functional magnetic resonance imaging (rs-fMRI) modality has gained widespread acceptance as a promising method for analyzing a variety of neurological and psychiatric diseases. It is established that resting-state neuroimaging data exhibit fractal behavior, manifested in the form of slow-decaying auto-correlation and power-law scaling of the power spectrum across low-frequency components. With this property, the rs-fMRI signal can be broken down into fractal and nonfractal components. The fractal nature originates from several sources, such as cardiac fluctuations, respiration and system noise, and carries no information on the brain’s neuronal activities. As a result, the conventional correlation of rs-fMRI signals may not accurately reflect the functional dynamic of spontaneous neuronal activities. This problem can be solved by using a better representation of neuronal activities provided by the connectivity of nonfractal components. In this work, the nonfractal connectivity of rs-fMRI is used to distinguish Alzheimer’s patients from healthy controls. The automated anatomical labeling (AAL) atlas is used to extract the blood-oxygenation-level-dependent time series signals from 116 brain regions, yielding a 116 × 116 nonfractal connectivity matrix. From this matrix, significant connections evaluated using the *p*-value are selected as an input to a classifier for the classification of Alzheimer’s vs. normal controls. The nonfractal-based approach provides a good representation of the brain’s neuronal activity. It outperformed the fractal and Pearson-based connectivity approaches by 16.4% and 17.2%, respectively. The classification algorithm developed based on the nonfractal connectivity feature and support vector machine classifier has shown an excellent performance, with an accuracy of 90.3% and 83.3% for the XHSLF dataset and ADNI dataset, respectively. For further validation of our proposed work, we combined the two datasets (XHSLF+ADNI) and still received an accuracy of 90.2%. The proposed work outperformed the recently published work by a margin of 8.18% and 11.2%, respectively.

## 1. Introduction

Alzheimer’s disease (AD) is a brain disorder that causes progressive deterioration in brain functions, commonly affecting memory function, the thinking process and behavior. Eventually, symptoms become severe enough to affect daily activities. Therefore, early diagnosis of Alzheimer’s offers a variety of advantages for diagnosed individuals and helps in their treatment planning. One of the effective methods to analyze brain functions is to observe the connectivity patterns of the brain. Brain connectivity refers to the way different brain units communicate with each other. Recent findings have shown that AD is strongly associated with alterations of network connectivity among different brain regions [1,2,3]. In Alzheimer’s disease patients, brain areas are poorly associated, and cognitive dysfunction is related to a reduced functional integration [3]. Hence, the brain connectivity patterns can be a useful biomarker to distinguish AD patients from normal controls (NC).

Different methods have been introduced over the years to characterize connectivity among the brain regions, and one of the early methods is the seed-based approach [4]. The seed-based approach chooses a brain region as the seed and finds the temporal correlation between the selected seed to the rest of the brain. Although known to be computationally simple and a more intuitive result analysis, the seed-based approach is dependent on the selection of seeds, making it vulnerable to bias. An application of seed-based connectivity for AD classification from normal controls was conducted in [5,6,7,8], considering the specific brain regions—posterior cingulate cortex, middle temporal gyrus, entorhinal cortex and hippocampus—that are highly affected by AD. The findings from these studies show that the brain connectivity in these regions carries the key features for the identification of AD from normal controls. However, seed-based analysis has the advantage of displaying the network of regions that are the most functionally related to the region of interest. This interpretation is simple and appealing to many experts. The fundamental constraint of this method is noise created by other structural spatial resting networks influenced by head movements or scanner-induced distortions.

In general, conventional methods for diagnosing AD and NC are developed using positron emission tomography (PET) and cerebrospinal fluid (CSF) [9]. The use of CSF as an AD biomarker is not economical and the interpretation of results is challenging and complex [10]. Recent years have seen a tremendous increase in AD-related research utilizing other brain imaging modality, including electroencephalography (EEG) [11,12], which suffers from a low spatial resolution due to the smaller number of electrodes used, functional magnetic resonance imaging (fMRI) and structural magnetic resonance imaging (sMRI) [13]. In comparison to EEG, with a better spatial resolution, fMRI signals quantify brain activity based on the changes in oxygenation, blood volume and flow. In contrast to the structural MRI, which mostly reflects the brain tissue information, the fMRI focuses on functional brain activities and provides a more direct measurement on the involvement of different brain regions in certain brain activities [14].

### 1.1. Alzheimer’s Disease Classification—Related Work

Early diagnosis of Alzheimer’s disease (AD) can be beneficial for the diagnosed individuals and their caregivers. Among the benefits is a better prognosis, which can facilitate earlier treatment and allow for the arrangement of specialized social care and counseling for the patients and their family members. For this reason, different methods were proposed over the years for the analysis and classification of AD patients from NC using either structural MRI, blood oxygen level-dependent (BOLD) time-series fMRI signals or a combination of brain imaging modality.

The Alzheimer’s Disease Neuroimaging Initiative (ADNI) database is one of the most commonly used datasets for the classification of AD, mild cognitive impairment (MCI) and normal controls (NC) [15,16,17,18]. In [16], Heung et al. developed a deep learning-based classification algorithm for the diagnosis of AD and MCI. The approach blends sparse regression models with a deep neural network, with the convolutional neural network (CNN) using the forecasts from various regression models as feedback for making final clinical decisions. The ADNI provided an MRI dataset of 805 subjects, and the work reported a 91.02% classification accuracy. In [17], Esmaeilzadeh et al. proposed a 3D CNN to address the issue of a small number of available labeled subjects, reporting a high dimensionality of neuroimaging data for the diagnosis of MCI/AD, with an accuracy of 94.1%. In another work, Jack Albright [18] suggested a neural network-based approach for forecasting the progression of Alzheimer’s disease with a 0.866 multi-class area under the curve (AUC), and the trained model was effective in assessing the progression of Alzheimer’s disease in patients who were cognitively stable at the start and in patients who had moderate cognitive dysfunction.

A technique based on structural and metabolic connectivity was used by Zheng et al. [15] to distinguish between AD and MCI patients. Using multi-modal images, both structural and metabolic connectivity was obtained, and MRI and positron emission tomography (PET) were used, which represent the high-order morphological and metabolic interactions in the network. The proposed method achieved a 79.37% accuracy in predicting MCI-to-AD progression and demonstrated the good potential of multi-modal connectivity biomarkers for early AD diagnosis. In [19], Castellazzi et al. proposed a method based on multiple regional metrics from rs-fMRI and diffusion tensor imaging (DTI) as input features to a classifier for the automatic identification of AD from vascular dementia (VD). The method was tested on 33 AD and 27 VD using an adaptive neuro-fuzzy inference system (ANFIS) and yielded a correct prediction rate of 77.33%. In another work, a tensor-based framework for rs-fMRI classification achieved an accuracy of 86% [20]. The proposed algorithm utilized a novel connectivity pattern and has boosted the classification of early-stage AD.

The analysis of the brain network is an effective way of defining brain topological organization, which has been extensively used in the investigation of mental disorders [15,21]. It is indicated in [21] by Frank et al. that functional connectivity can be used to classify Alzheimer’s disease and to identify its distinguishing features. Another significant measure to observe and visualize brain functions is an efficient imaging modality to evaluate how structurally separated and functionally specialized brain networks are linked, especially using rs-fMRI [22], which reflects spontaneous BOLD time-series signal fluctuations when a subject is not undertaking any explicit tasks. The use of BOLD time series rs-fMRI signals and deep learning techniques for the diagnosis of AD is reported in [22,23]. In [22], Ju et al. used the functional connectivity of rs-fMRI as an input to an autoencoder network for distinguishing normal ageing from MCI, which is an early stage of AD. Wang et al. used permutation entropy to investigate the complexity of rs-fMRI signals in MCI and AD patients [23]. From MCI to AD, the permutation entropy has been shown to decrease. This finding shows that rs-fMRI signal complexity analyses can be used to characterise cognitive impairments in MCI and AD.

Several studies have shown that global artifacts coming from motion and other physiological factors influence brain connectivity [24,25,26], and several approaches have been undertaken to decrease the effects of these artifacts. Global signal regression is utilized in [24] to reduce global artifacts caused by motion and respiration. The proposed regression method strengthens the associations between the functional connectivity of resting state signals and most behavioral measures of young healthy adults. A regression technique was proposed by Rasmus et al. to remove breathing variation-related fluctuations from neuronal activity using a simultaneous recording of heartbeat signals [25]. Monofractal and multifractal dynamics in fMRI have been studied by Jang et al. [26], and Wink et al. [27] introduced a feature extraction method for task-based fMRI recording in classifying fMRI volumes using a deep neural network.

### 1.2. Fractal Behavior of rs-fMRI Signals

**Figure 1:**
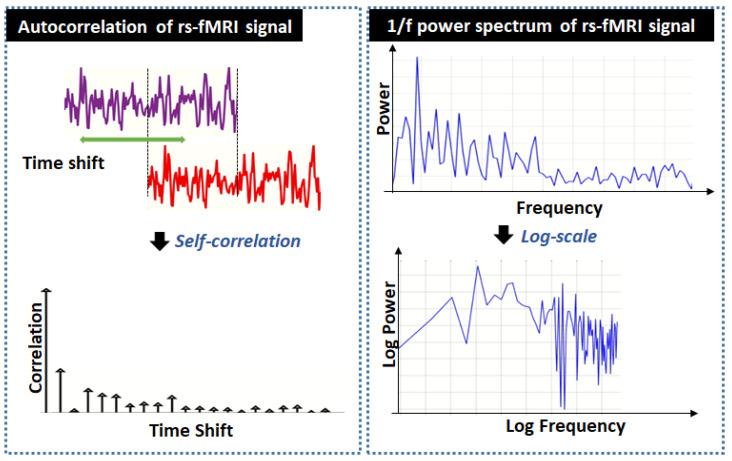
Autocorrelation and power spectrum fractal properties of rs-fMRI signals.

One of the main objectives of resting-state neuroimaging is to detect the physiological mechanisms of resting-state brain imaging data accurately. However, this is not straightforward as non-neuronal physiological influences have a significant effect on the resting state signals and these influences need to be taken into consideration in the development of the classification algorithm for AD. It is established in [28,29,30,31,32,33,34,35] that resting-state fMRI signal follows fractal behavior, also known as long-range dependence, and they exhibit self-similarity and power-law scaling properties in the time and frequency domain, respectively. The self-similarity property manifests in the form of a slow decaying autocorrelation of the resting-state fMRI time-series signal. In the frequency domain, fractal properties exhibit in the form of a 1/f power spectrum. This concept is illustrated in Figure 1.

In neuroimaging signals, the fractal nature may originate from several sources, such as cardiac fluctuations [36], respiration [25], vascular changes [37] and system noise. It is established that fractal behavior will affect the functional connectivity [35,38,39]; hence, extracting fractal-free signal connectivity matrices is paramount for the accurate classification of AD using rs-fMRI signals. This fractal behavior can also be observed in time-series prediction and optimization [40,41]. The connectivity matrix of fractal-free signals computed via the wavelet transform of the long memory process of rs-fMRI signals is known as nonfractal connectivity [35]. It provides a better depiction of the brain’s neuronal activities as it cancels the effect of functional connectivity from fractal behavior. Nonfractal connectivity is a correlation of short-term memory signals and is independent of fractal behavior.

**Figure 2:**
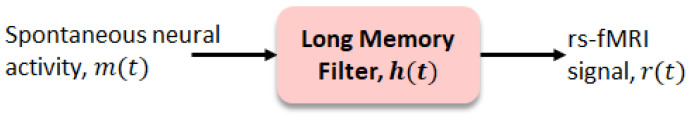
Linear FIP model of rs-fMRI signals.

The concept of fractal and nonfractal functional connectivity of rs-fMRI was introduced in 2012 by You et al. [35] and was implemented in the analysis of an rs-fMRI recording of a rat. The paper provides a representation of the fractal behavior of neuroimaging signals based on the fractionally integrated process (FIP) model. Fundamentally, the neuroimaging signal is interpreted in the FIP model as the output of a long-memory (LM) filter whose input is a nonfractal signal. In other words, through long memory filtering, a nonfractal signal is translated into a neuroimaging signal with fractal behavior, as illustrated in Figure 2. To date, the nonfractal functional connectivity has not been used to distinguish AD from NC subjects.

The proposed research makes the following contributions and is summarized as follows. This study uses nonfractal connectivity to create a classification algorithm for AD vs. NC individuals. The method is projected to outperform standard correlation in classification because nonfractal connectivity gives a more accurate depiction of the brain’s neural activity [35]. The connectivity of 116 AAL brain areas is estimated, yielding a 116 times 116-connectivity matrix per participant. p-value analysis is used to find significant connections, which will be fed into machine learning classifiers. A comparison between Pearson correlation and fractal connectivity is made on multiple classifiers to demonstrate the excellent classification performance provided by nonfractal connectivity. It outperformed the fractal and Pearson-based connectivity techniques by 16.4% and 17.2%, respectively. The proposed method is unique because it uses the nonfractal functional connectivity of fractal-free rs-fMRI data to distinguish between AD and NC subjects. This is the first time fractal-free rs-fMRI data have been used to distinguish Alzheimer’s patients from healthy controls. The value of the proposed approach is demonstrated using two independent datasets with distinct acquisition protocols, proving the high performance of the nonfractal connectivity measure. The nonfractal method has been used in the analysis of rs- fMRI data to date [35,38,39], but no investigation into neurological disorder classification has been performed.

The rest of the paper is structured as follows. Section 2 presents the long memory process for modeling the fractal nature of rs-fMRI data. Methods of the investigation, details on the dataset and the principles of the wavelet-based fractal analysis of brain connectivity are described in Section 3. In Section 4, results of the investigation on rs-fMRI in classifying the rs-fMRI signal for the detection of AD from HC are presented, including the statistical analysis, feature selection and performance of the classification algorithm tested on several machine learning classifiers.

## 2. Long Memory Model of rs-fMRI Signals

In the following section, we cover univariate and multivariate fractionally integrated processes (FIPs), which include fractionally integrated noise (FIN), fractional Gaussian noise (FGN) and the auto-regressive fractionally integrated moving average (ARFIMA).

### 2.1. Univariate Case

The output of the linear LM filter is r(t), and it is a real-valued discrete process of length *N*. Then, the input to the LM filter is the spontaneous neural activity that exhibits short-term memory, where m(t), which has spectral density $M_{m}\left(f\right)$, is

(1) $$m\left(t\right)={\left(1-P\right)}^{a}r\left(t\right),$$

where a∈R is the Hurst exponent calculated from wavelet-based fractal estimation and the back-shift operator is defined as *P*. The *a* parameter controls the fractal behavior, where, if 0<a<1/2, the r(t) is anti-persistent and exhibits short memory, whereas if a>1/2, the r(t) is persistent and exhibits long memory. On the other hand, the white noise process will have a=0 [42,43,44].

In essence, the output of the linear LM filter, r(t), which is the convolution of the spontaneous neural activity, m(t), with the filter impulse response, h(t), given as

(2) $$r\left(t\right)=\sum_{\tau =0}^{\infty }h\left(\tau \right)m\left(t-\tau \right),$$

where

(3) $$h\left(t\right)\frac{a\Gamma (a+t)}{\Gamma (a+1)\Gamma (t+1)},$$

If $-\frac{1}{2}<a<\frac{1}{2}$, the spectral density of r(t) can be given as

(4) $$S_{r}\left(f\right)={\left|1-e^{-jf}\right|}^{-2a}S_{m}\left(f\right).$$

Here, the term $|1-e^{-jf}{|}^{-2a}$ in Equation (4) represents the fractal component of r(t), whereas the $S_{m}\left(f\right)$ represents the nonfractal component of r(t) [35].

### 2.2. Multivariate Case

The univariate model of long memory can be enhanced to a multivariate case. A real-valued *v*-vector process R(t) is given by

(5) $$\left(\begin{array}{ccc} {\left(1-P\right)}^{a_{1}} & \; & 0\\ \; & \ddots & \\ 0 & \; & {\left(1-P\right)}^{a_{v}} \end{array}\right)\left(\begin{array}{c} r_{1}\left(t\right)\\ \vdots \\ r_{v}\left(t\right) \end{array}\right)=\left(\begin{array}{c} m_{1}\left(t\right)\\ \vdots \\ m_{v}\left(t\right) \end{array}\right),$$

where $M\left(t\right)=(m_{1}\left(t\right),\cdots ,m_{v}\left(t\right))$ represents a multivariate stationary process and its spectral density $S_{u}\left(f\right)=\left[S_{x,y}\left(f\right)\right]$ is confined within (−π,π). For $-\frac{1}{2}<a_{l}<\frac{1}{2}$, the spectral density of M is given as

(6) $$S_{u}\left(f\right)=\psi \left(f\right)S_{m}\left(f\right)\psi^{*}\left(f\right),$$

where

(7) $$\psi \left(f\right)=\left(\begin{array}{ccc} {\left(1-e^{jf}\right)}^{-a_{1}} & \; & 0\\ \; & \ddots & \\ 0 & \; & {\left(1-e^{jf}\right)}^{-a_{v}} \end{array}\right).$$

Providing, $0<a_{l}<\frac{1}{2}$ for 1≤l≤v, R(t) is considered to be a stationary long-memory process having memory parameter $a=[a_{1},\cdots ,a_{v}]$. Assuming M(t) to be a vector of the auto-regressive moving average (ARMA) process, R(t) eventually becomes a multivariate ARFIMA process. However, on the other hand, if M(t) is a vector i.i.d random variable, i.e.,

(8) $$M\left(t\right)\;\overset{i.i.d.}{\;∼}\;N\left(0,\sum_{m}\right).$$

R(t) becomes a multivariate FIN. Now, the cross-spectral density of $r_{x}\left(t\right)$ and $r_{y}\left(t\right)$ is given as

(9) $$S_{x,y}\left(f\right)=\eta_{x,y}{\left(1-e^{jf}\right)}^{-a_{x}}{\left(1-e^{-jf}\right)}^{-a_{y}},$$

where $\eta_{x,y}$ is the (x,y)-th element of $\sum_{m}$.

## 3. Methodology

**Figure 3:**
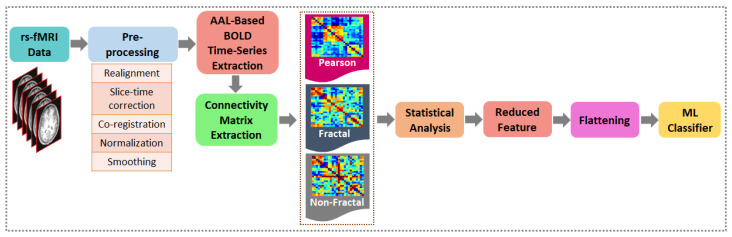
The proposed method of investigation for development of AD vs. NC classification algorithm using wavelet-based fractal analysis of rs-fMRI signals.

In Figure 3, the proposed AD vs. NC classification algorithm employing nonfractal connectivity of rs-fMRI data is shown. Initially, the raw data were pre-processed using a conventional method. The AAL atlas was then used to extract time series from 116 regions, followed by the generation of connectivity matrices. Before being fed into the machine learning (ML) classifier, the connectivity matrices must be statistically analyzed to minimize the feature vector. The evaluation of the nonfractal classification algorithm was compared to fractal connectivity and Pearson correlation as a baseline comparison. A 10-fold cross-validation framework was used for parameter optimization and classification.

### 3.1. Description of Dataset

The rs-fMRI datasets obtained from three sites were used in this study, abbreviated as XH [45], SLF [46] and ADNI [47]. The demographic and physiological information of the subjects for each site is summarized in Table 1 and the data acquisition protocol of the MRI scanner is given in Table 2.

The first one is the XH dataset from the neuropsychological research facility at Xuanwu Hospital, Beijing, China [45], having a total of 56 subjects, comprising 21 normal controls (NC) and 35 AD subjects. Among the 21 healthy volunteers, there were 7 males and 14 females, with age of 65.0 ± 8.1 years, whereas, among the 35 AD, there were 17 males and 18 females, with age of 65.8 ± 8.3 years. For a balanced class size of AD and NC, 31 AD and 21 NC were selected from the XH, with an additional 10 NC from the second dataset from Santa Lucia Foundation (SLF) [46], making the total number of NC 31. The combined rs-fMRI dataset of XH and SLF are denoted as XHSLF dataset in the Result and Discussion section.

The third dataset was obtained from the Alzheimer’s Disease Neuroimaging Initiative (ADNI) database [47] consisting of 60 subjects, with 30 from AD (average age 72.81 years) and 30 from NC (average age 75.25 years). Patients with AD have MMSE score of 20–26 and CDR of 0.5–1, whereas NC patients have MMSE score of 24–30 and a CDR of 0. The idea behind taking 30 subjects from each class for ADNI is based on the equal number of time points. Basically, ADNI contains fMRI data with different number of time points, i.e., 140, 197, 200, etc. We took those subjects that had 140 time points. However, other subjects with different number of time points can also be considered in the future studies.

### 3.2. Data Pre-Processing

For the analysis of fMRI data, pre-processing is necessary. These steps are crucial in making the analysis legitimate and greatly improving the ability of the subsequent analyses, as it removes unwanted artifacts and transforms the data into a standard format. Data were pre-processed using the connectivity toolbox (CONN) toolbox in the following order: realignment, slice time correction, co-registration, normalization and smoothing. After image pre-processing, time-series were extracted using Data Processing & Analysis for (Resting-State) Brain Imaging (DPABI) toolbox [48].

### 3.3. BOLD Time-Series Signals Extraction

After following the standard procedure of pre-processing, the connectivity matrix was computed using time-series signals from 116 regions described by the AAL atlas. With a signal length of 160 samples, the size of the extracted signal for each subject was 160-sample × 116-region.

### 3.4. Functional Connectivity of rs-fMRI Signals

Based on wavelet fractal analysis, 2 types of connectivity were extracted, namely fractal and nonfractal, as the feature vector for the classification problem. In addition, for base comparison, Pearson correlation was also considered for the classification of AD vs. NC. The detailed calculation of Pearson correlation and the principle of fractal and nonfractal connectivity are given in the subsequent sections.

#### 3.4.1. Pearson Correlation Coefficient

A standardized measure of covariance between two variables is the Pearson correlation coefficient [49]. The Pearson correlation between two variables is calculated as follows:

(10) $$P_{A,B}=\frac{\mathrm{cov}(A,B)}{\sigma_{A}\sigma_{B}},$$

where $P_{A,B}$ shows the Pearson correlation between time series *A* and *B*, cov is the covariance and $\sigma_{A}$ and $\sigma_{B}$ are the standard deviations of *A* and *B*, respectively. The covariance can be calculated by

(11) $$\mathrm{cov}\left(A,B\right)=E[\left(A-\mu_{A}\right)\left(B-\mu_{B}\right)],$$

where E[.] is the expectation operator and $\mu_{A}$ and $\mu_{B}$ are the mean of *A* and *B*, respectively. Equation (10) can be re-written as

(12) $$P_{A,B}=\frac{E[\left(A-\mu_{A}\right)\left(B-\mu_{B}\right)]}{\sigma_{A}\sigma_{B}}.$$

#### 3.4.2. Wavelet Analysis for Fractal Connectivity

The Pearson correlation provides a measure of similarity between raw BOLD time-series signals of different brain regions. It is one of the early measures on brain signals’ functional connectivity. Using the multivariate long memory model presented in Section 2, two new concepts of functional resting-state connectivity between brain regions—fractal connectivity and nonfractal connectivity—were introduced [35]. The former is defined as the asymptotic wavelet correlation, meaning as the wavelet scale approaches to infinity, whereas the latter is described as the short memory covariance of BOLD time-series signals from a pair of brain regions.

Consider R(t) to be the multivariate FIN process, representing the BOLD time-series signal of interest, with the memory parameter *a*, and M(t) to be a short memory function of R(t) given in Equation (5). The nonfractal connectivity of $r_{x}\left(t\right)$ and $r_{y}\left(t\right)$ is described as

(13) $$A_{x,y}=\frac{\eta_{x,y}}{\sqrt{\eta_{x,x}\eta_{y,y}}},$$

where $\eta_{x,y}$ represents the covariance of spontaneous neural activity signals, $m_{x}\left(t\right)$, and $m_{y}\left(t\right)$; that is, $\eta_{x,y}$: = $E[m_{x}\left(1\right)m_{y}\left(1\right)]$.

#### 3.4.3. Wavelet Analysis for Nonfractal Connectivity

By means of the discrete wavelet transform (DWT), the variance of a discrete time series can be broken down across several frequency bands. Consider the wavelet coefficient of the *p*-th process $r_{p}\left(t\right)$ at scale *q* and time point *s* to be $V_{p}\left(q,s\right)$. The wavelet covariance is given as $\upsilon_{x,y}\left(q\right):\;=\;cor\left(V_{x}\left(q,s\right),V_{y}\left(q,s\right)\right)$ at scale *q*. In addition, the coefficient of the wavelet of an FIP is covariance stationary at scale *q*, and $\upsilon_{x,y}\left(q\right)$ is independent of time *t*.

(14) $$B_{q}\left(f\right)\approx \left\{\begin{array}{cc} 2^{q} & \mathrm{for}\;2\pi /2^{q+1}\leq \left|f\right|\leq 2\pi /2^{q}\\ 0 & \;\;\;\mathrm{otherwise} \end{array}\right.$$

Finally, the wavelet covariance of $r_{x}\left(t\right)$ and $r_{y}\left(t\right)$, at scale *q* is associated with the following cross-spectral density [50] of the wavelet coefficients at scale *q* and the BOLD time-series signals:

(15) $$\upsilon_{x,y}\left(q\right)=2\pi \int_{-\pi }^{\pi }B_{q}\left(f\right)S_{R}\left(f\right)df.$$

The wavelet correlation at scale *q* and time point *s*, $\delta_{x,y}\left(q\right)$: = cor$\left(V_{x}\left(q,s\right),V_{y}\left(q,s\right)\right)$ is written as

(16) $$\delta_{x,y}\left(q\right)=\frac{\upsilon_{x,y}\left(q\right)}{\sqrt{\upsilon_{x}\left(q\right)\upsilon_{y}\left(q\right)}}.$$

**Theorem** **1**  (Asymptotic wavelet covariance). *Assume that R(t) is a multivariate FIN process that is i.i.d random variable meeting the criteria, as in Equation (8). Then, the asymptotic wavelet covariance of $r_{x}\left(t\right)$ and $r_{y}\left(t\right)$ computed at scale q→∞ is given by*

(17) $$\upsilon_{x,y}\left(q\right)\approx \eta_{x,y}\beta_{x,y}2^{q(a_{x}+a_{y})}\;\mathrm{as}\;q\to \infty ,$$

*where*

(18) $$\beta_{x,y}:=2\cos\left(\frac{\pi }{2}\left(a_{x}-a_{y}\right)\right)\frac{1-2^{a_{x}+a_{y}-1}}{1-a_{x}-a_{y}}{\left(2\pi \right)}^{-a_{x}-a_{y}}.$$

From (16) and (17), the wavelet correlation of $r_{x}\left(t\right)$ and $r_{y}\left(t\right)$ as the scale *q* approaches *∞* is the fractal connectivity of the multivariate FIN, given as

(19) $$\delta_{x,y}\left(q\right)\to \delta_{x,y}^{\infty }:=A_{x,y}\frac{\beta_{x,y}}{\sqrt{\beta_{x,x}\beta_{y,y}}}\;\mathrm{as}\;q\to \infty .$$

In essence, the fractal connectivity of $r_{x}\left(t\right)$ and $r_{y}\left(t\right)$ is the asymptotic wavelet correlation, $\delta_{x,y}^{\infty }$. Notably, both nonfractal connectivity in Equation (19) and fractal connectivity in Equation (13) require estimation of memory parameters $a_{x}$ and $a_{y}$, as well as calculation of short memory covariance, as in Equation (13). Method of estimating the Hurst exponent and short memory covariance matrix of a multivariate FIN via the univariate maximum likelihood method was proposed by You et al. in [35]. The likelihood function for memory parameter $a_{x}$ is given by

(20) $$L\left({\hat{a}}_{x},{\hat{\eta }}_{x}|r_{x}\left(t\right)\right):=\frac{1}{{\left(2\pi \right)}^{N/2}{\left|\Sigma_{x}\right|}^{1/2}}e^{-r^{T}\Sigma_{x}^{-1}r/2},$$

where the matrix $\Sigma_{x}$ denotes the covariance matrix of $r_{x}\left(t\right)$. The optimal memory parameter ${\hat{a}}_{x}$ is obtained via a minimization problem derived from Equation (20) with respect to ${\hat{a}}_{x}$ [35]. The estimation of short-memory covariance for nonfractal connectivity is also obtained by using the linearity of wavelet covariance over scales, given as

(21) $${\hat{\eta }}_{x,y}=\frac{2^{{\hat{c}}_{x,y}-1}}{B_{x,y}\cos\left(\frac{\pi }{2}\left(a_{x}-a_{y}\right)\right)}{\left(2\pi \right)}^{a_{x}+a_{y}},$$

where

(22) $${\hat{c}}_{x,y}=\frac{1}{Q}\sum_{q=1}^{Q}\left[\log_{2}{\hat{\upsilon }}_{x,y}\left(q\right)-\left(a_{x}+a_{y}\right)q\right],$$

(23) $$B_{x,y}:=\frac{1-2^{a_{x}+a_{y}-1}}{1-a_{x}-a_{y}}.$$

**Figure 4:**
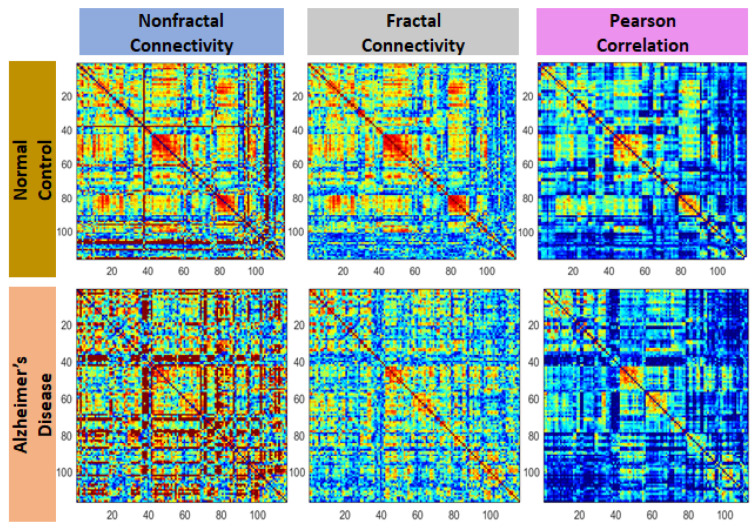
Samples of nonfractal, fractal and Pearson connectivity matrices from normal control (NC) and an Alzheimer’s disease (AD) subject extracted using the AAL-based BOLD time series signals.

Figure 4 shows a sample of 116 × 116 AD and NC connectivity matrices for nonfractal, fractal and Pearson.

### 3.5. Statistical Analysis, Feature Reduction and Flattening of Functional Connections

The 160×116 time-series signal of one subject will result in a total number of 116 × 116 = 13,456 connections. For accurate classification between the AD and NC, only highly meaningful features were selected using the one-way analysis of variance (ANOVA) of the connectivity values and expressed in terms of *p*-value. Apart from reducing the length of the input feature vector to ML classifiers, this will also reduce the computational cost of modeling and improve the performance of the model. Since the conventional ML classifiers only accept 1D input type, the reduced feature vector is flattened prior to being input to ML classifiers.

### 3.6. ML Classifier

The final stage in the classification process is to choose a classifier. The support vector machine (SVM) is a supervised machine learning technique that performs well even when feature vectors have many dimensions. The SVM separated the classes using a line in 2D cases, and a plane in higher dimensions using a constraint optimization problem [51]. When the classes were not linearly separable, the feature vector was transformed into a new feature space and used to derive the decision boundary in the original feature space using a kernel mapping method. A linear kernel function was used in this study.

The equation of a linear hyperplane is given as follows

(24) w·x+b=0

where *w* is defined as a normal to the hyperplane, the feature vector is given by *x* and bias is given by *b*. Values for *b* and *w* are obtained by SVM from the training data. For a classifier having a decision boundary, it can be written as:

(25) $$x_{i}\cdot w+b\geq 1\;\mathrm{for}\;y_{i}=+1$$

(26) $$x_{i}\cdot w+b\leq -1\;\mathrm{for}\;y_{i}=-1$$

Combining Equations (25) and (26) we obtain

(27) $$y_{i}\left(x_{i}\cdot w+b\right)-1\geq 0\;\forall \;i$$

The constraint in Equation (27) is that all training data must lie on either side of the support hyperplane. Support vectors are the points that are closest to the separating hyper-plane.

Other classifiers, including KNN, decision trees and bagged trees, were also tested in addition to SVM, and the findings of the top four classifiers are reported in the result section. The best machine learning hyperparameters for SVM, KNN, decision trees and bagged trees were chosen using Bayesian optimization, with cross-validation loss as the objective function.

### 3.7. Performance Evaluation

Let AD be the positive and NC be the negative class. The following performance measures were calculated using the confusion matrix, as shown below.

(28) $$\mathrm{Sensitivity}=\frac{TP}{TP+FN}$$

(29) $$\mathrm{Specificity}=\frac{TN}{TN+FP}$$

(30) $$\mathrm{Accuracy}=\frac{TP+TN}{TP+TN+FP+FN}$$

(31) $$\mathrm{Precision}=\frac{TP}{TP+FP}$$

(32) $$\mathrm{False}\;\mathrm{Positive}\;\mathrm{Rate}\;\left(\mathrm{FPR}\right)=\frac{FP}{TN+FP}$$

True positive is TP, true negative is TN, false positive is FP and false negative is FN. In addition to the above metrics, we use the area under the curve (AUC)–receiver operating characteristics (ROC) curve to visualise the classifier’s performance. The ROC curve is a probability plot of the true positive rate (TPR), commonly called sensitivity, versus the false positive rate (FPR). The higher the AUC, the better the model distinguishes between AD and NC, implying a high level of separability.

## 4. Result and Discussion

The performance of the three connectivity matrices—nonfractal, fractal and Pearson—in classifying AD over NC is evaluated in this section. Results from the best performance classifiers, generated using a 10-fold cross-validation framework, are presented for comparison.

The two datasets, XHSLF and ADNI, are independently evaluated, since the data were recorded using different protocols and have slightly different demographics. Data from 62 subjects from the XHSLF dataset and 30 subjects from the ADNI dataset are used to generate the 116×116 connectivity matrices for each subject, where only the significant connections with *p* ≤ 0.05 are selected using *p*-value analysis as the input feature to ML classifiers.The coding and training of the classifier models were run on MATLAB 2020a on a ninth generation Intel i7-9700 Processor CPU operating at 3 GHz frequency.

### 4.1. Statistical Analysis

**Figure 5:**
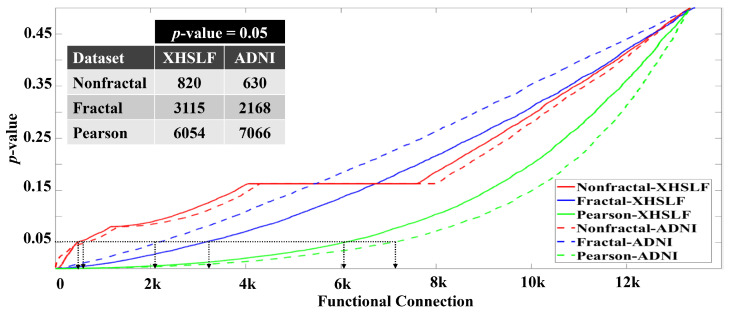
Plot of p-value of 13,456 connections of XHSLF and ADNI datasets arranged in increasing order for nonfractal, fractal and Pearson correlation. The number of significant connections, obtained at p=0.05, for each connectivity measure is listed in the inset table.

To distinguish between AD and NC, the *p*-value analysis is used to examine the significance connections for the three connectivity matrices: the nonfractal, fractal and Pearson correlation. Figure 5 shows the *p*-values of 13,456 connections for the nonfractal, fractal and Pearson correlation for the datasets XHSLF and ADNI. Using the confidence level of 95%, the total informative connection of XHSLF is 820, 3115 and 6054 whereas, for the ADNI dataset, the values are 630, 2168 and 7066, for the nonfractal, fractal and Pearson correlation, respectively. These functional connections will be used as the input vectors to ML classifiers. Relative to nonfractal, the fractal and Pearson correlation contains more significant connections, producing higher dimensional feature vectors.

### 4.2. Classification of AD vs. NC Based on Nonfractal Connectivity

The results presented in this section are arranged in the following manner. Firstly, the classifications based on nonfractal, fractal and Pearson connectivity are experimented on using several classifiers, and evaluated in terms of accuracy. Secondly, using the best classifier as determined from the first experiment, apart from the accuracy, the classification model is further evaluated, in terms of sensitivity, specificity, precision, FPR and AUC. Both experiments are conducted using a 10-fold cross-validation framework.

#### 4.2.1. Selection of the Best ML Classifier

In the first experiment, after testing with several classifiers, the performances of the three best individual classifiers and the best ensemble classifier are listed in Table 3. The length of the feature vector for nonfractal, fractal and Pearson connectivity is based on the length of the reduced feature, as discussed in Section 4.1 and shown in Figure 5.

The parameters for the classifiers are as follows: The KNN classifier is trained using 11 neighbors and the cosine distance metric. The decision tree employed Gini’s diversity index split criteria. The bagged tree employed 30 learners, with the weighted average rule as the basis for the choice. Finally, employing the linear kernel function, the best performance of the SVM was achieved.

Many classifiers were tested in the search for the best one. Here, only classification accuracy values for the three best non-ensemble and the best ensemble methods are presented in Table 3. Clearly, for most of the classifiers and for both datasets, nonfractal connectivity gives a better classification accuracy than fractal and Pearson. The best performance by nonfractal connectivity is 90.3%, whereas fractal and Pearson connectivity are at 82.3% and 72.6%, respectively. Since the SVM gives the best average performance for nonfractal connectivity across the two datasets, the subsequent investigations will be based on the SVM classifier.

#### 4.2.2. Evaluation of Significant Functional Connections

To further substantiate the good performance of the reduced nonfractal connectivity over fractal and Pearson connectivity, the SVM classifier is tested with the same length of the feature vector, equal to the number of nonfractal functional connections. This means that, for the XHSLF dataset, the length of the nonfractal, fractal and Pearson connectivity is set at 820, and, for the ADNI dataset, it is set at 630.

The classification accuracy using the SVM classifier at a fixed length of the feature vector is reported in Table 4. As expected, the nonfractal-based approach consistently gives the highest accuracy compared to the fractal and Pearson approaches. Notably, the highest accuracy of the nonfractal approach is recorded using 10-fold cross validation, at 90.3%, which is approximately 1.24 and 1.75 times higher than the fractal and Pearson approaches, respectively, for the XHSLF dataset, and 1.31 times higher for the ADNI dataset.

The *p*-value of the top 820 connections of the XHSLF dataset for fractal and Pearson connectivities is at 0.007 and 0.009, respectively, whereas, for the ADNI dataset, the *p*-value of the top 630 connections for fractal and Pearson connectivites is at 0.0012 and 0.0006, respectively. This means that, at the same length as the nonfractal connection, the significant connections of fractal and Pearson have higher confidence levels. However, even with higher confidence levels, the fractal and Pearson connectivities were not able to result in a good accuracy like the nonfractal connectivity. This is possibly due to the better representation of the brain’s neuronal activity by the nonfractal connectivity, relative to the fractal and Pearson connectivities.

#### 4.2.3. Classification of AD vs. NC Using SVM

In this section, the results of a detailed evaluation using the best classifier, SVM, based on nonfractal, fractal and Pearson connectivity measures are presented. The length of the feature vector for each connectivity measure and dataset are determined, as in Section 4.1.

The detailed evaluation of the classification algorithm using nonfractal connectivity and SubEn includes assessments on accuracy, sensitivity, specificity, precision, FPR and AUC. Using a 10-fold framework, the results of the assessment are presented in Table 5. Here, only the significant connections are used as the input vectors of the classifier. The SVM with a linear kernel is used as it performs well with high-dimensional functional connectivity data.

**Figure 6:**
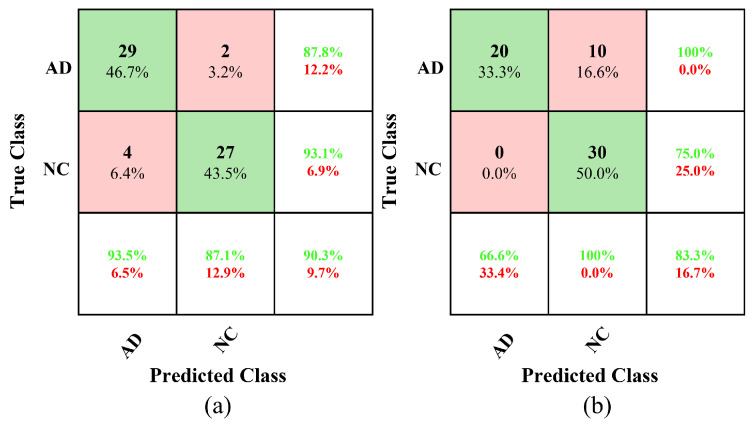
Confusion matrix for classification of AD vs. NC using nonfractal connectivity of rs-fMRI as input feature vectors to SVM classifier for dataset (**a**) XHSLF and (**b**) ADNI.

For the XHSLF dataset, the nonfractal connectivity achieves the highest accuracy of 90.3%, with an 87.87% sensitivity and 93.1% specificity, an AUC value of 0.98 and an FPR of around 6.89%, demonstrating its overall good performance relative to benchmark methods, and fractal and Pearson connectivity. For the ADNI dataset, the performance of nonfractal connectivity is slightly lower but maintained at 83.3% accuracy. Apart from the performance measures presented in Table 5, the plots of the confusion matrix for both the XHSLF and ADNI dataset are also shown in Figure 6.

**Figure 7:**
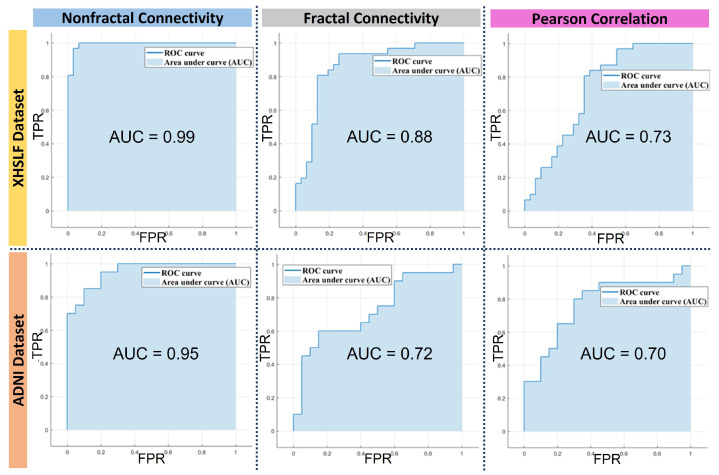
ROC curve and AUC for classification of AD vs. NC using functional connectivity of rs-fMRI as input feature vectors to SVM classifier for dataset XHSLF and ADNI.

With nonfractal AUC values of 0.99 and 0.95 for the XHSLF and ADNI datasets, respectively, this indicates that the model yields a high true positive rate and low false positive rate. The closer the AUC value is to 1, the better the model isat predicting AD as AD and NC as NC. For further demonstration on the excellent performance of the classification using nonfractal connectivity, the receiver operator characteristics (ROC) curve is generated for the XHSLF and ADNI datasets, as shown in Figure 7. The ROC shows the relationship between sensitivity and specificity, as illustrated in the ROC curve analysis, resulting in an area under the curve (AUC) of 0.99 for the nonfractal, 0.86 for the fractal and 0.73 for the Pearson correlation for the XHSLF dataset. Similarly for the ADNI dataset, an AUC for the nonfractal is 0.95, 0.72 for the fractal and 0.77 for the Pearson correlation. The higher value of AUC for the nonfractal connectivity indicates the excellent classification performance of the proposed classification method.

### 4.3. Investigation on the Proposed AD Classification Using XHSLF+ADNI Dataset

**Figure 8:**
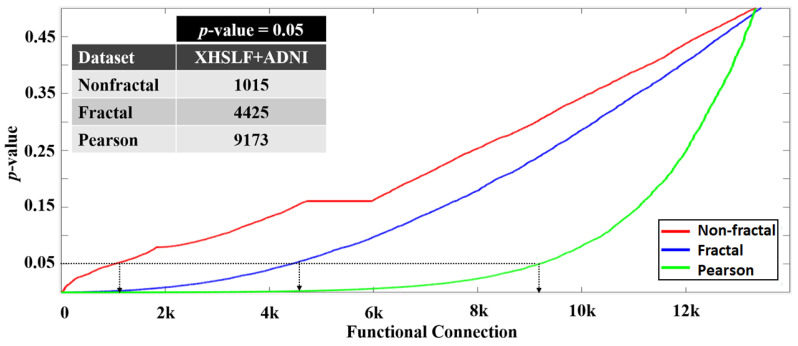
Plot of p-value of 13,456 connections of dataset XHSLF+ADNI arranged in increasing order for the nonfractal, fractal and Pearson correlation. The number of significant connections, obtained at p=0.05, for each connectivity measure is listed in the inset table.

The proposed approach is tested using a combined XHSLF and ADNI dataset with 122 subjects in this section. The combined dataset resulted in 61 subjects in each AD and NC class. Using *p*-value analysis, significance connections for the nonfractal, fractal and Pearson connectivities are determined at a 95% confidence level. The plot of *p*-values for 13,456 connections for the nonfractal, fractal and Pearson correlation for XHSLF+ADNI is shown in Figure 8. The number of significance connections for the nonfractal, fractal and Pearson correlation follows a similar trend as in Section 4.1 for XHSLF and ADNI. Specifically, relative to the nonfractal, the fractal and Pearson correlation contains more significant connections, producing higher dimensional feature vectors as input vectors to ML classifiers.

The proposed nonfractal-connectivity-based method obtained the highest accuracy of 90.2% using KNN with a linear kernel. However, the two other methods, namely the fractal and Pearson connectivity, achieved the highest accuracy of 73.8% with KNN and 73% with linear SVM, respectively. An evaluation was conducted in terms of sensitivity, specificity, accuracy, precision, FPR and AUC using different ML classifiers, as presented in Table 6. For the detailed evaluation, the findings based on nonfractal, fractal and Pearson connectivities are presented in Table 7. It can be seen that, when the number of subjects was increased to 61 per class, it still maintained a good classification accuracy of >90%. In fact, this value outperformed the two benchmark approaches, surpassing them by a margin of 16.4% and 17.2%, respectively.

Notably, the rs-fMRI signal is highly affected by several factors mentioned earlier. The oscillations and disruptions created by these factors affect the functional connectivity of the brain. They failed to give the pure neuronal brain activity, so the already available approaches do not reach a good classification accuracy, such as seed-based and ICA-based approaches. The signal that is not free from fractal behavior represented a poor accuracy, and the Pearson-based method also failed to perform effectively as the rs-fMRI signal contains the oscillations and noise. Moreover, it can be observed that the proposed method showed promising results on the individual and combined dataset, showing the excellent potential of the proposed method.

### 4.4. Comparison with Related Works

In Table 8, the findings of this study are compared to previous studies in terms of their accuracy, sensitivity and specificity. In Table 8, it is clear that, for the XHSLF dataset, the nonfractal approach outperformed the previous methods by Frank de Vos et al. [21] by 16.16% in terms of classification accuracy. For the AUC comparison, it surpassed the method by a margin of 0.14, respectively. However, the performance of the method for ADNI dataset is 4.3% higher than Frank de Vos et al. [21]. The lower accuracy for the ADNI dataset may arise from the lower number of subjects than that of the XHSLF dataset.

Furthermore, the work by Frank de Vos et al. [21] used 31 different features comprising functional connectivity matrices of several brain networks and fast eigenvector centrality mapping of the amplitude of low frequency fluctuations (ALFF) as an input vector to logistic regression for the classification of AD from NC. Since the method combines the feature vector from each resting state measure, the length of the combined feature vector is very large and causes a low classification accuracy.

Another machine learning method for the early diagnosis of Alzheimer’s disease was proposed by Kasani et al. [52], which was based on correlation of the demographic and neuropsychological information. Their reported accuracy of 82.75% for AD vs. NC classification was achieved using a bagging technique. The correlation of the demographic and neuropsychological information may not provide enough information for more promising results in AD classification.

In addition to this, the recently published work proposed by Zhu et al. [53] used seed correlation for the diagnosis of AD and showed that the functional connectivity between the hippocampus and other brain areas is altered in AD. Their proposed method achieved an accuracy of 82.02% for the classification of AD from NC subjects. The seed-based method suffers from the selection of the seed, which requires prior information and may lead to biases, hence not giving a good classification rate.

Overall, the proposed method outperformed the recently published work by 11.2% [21], 7.45% [52] and 8.18% [53], respectively.

## 5. Conclusions

This study uses the connectivity of nonfractal components of rs-fMRI signals to develop a classification method for AD vs. NC individuals. This is primarily in order to use the fact that nonfractal connectivity better represents spontaneous neural activity. In essence, nonfractal connectivity excludes the fractal components originated from system noise and other human physiological systems, such as respiratory functions, giving more accurate features of the brain signal for classifiers to learn in the discriminating of AD from normal control subjects. As a result of the limited number of training samples relative to a large number of features (116 × 116 = 13,456), the development of the classification algorithm can be challenging. Using significant connectivity values selected based on *p*-value analysis, out of 13,456 connections, 820 for the XHSLF dataset and 630 for the ADNI dataset are selected as the input of a SVM classifier. Our experimental results indicate that the nonfractal-based method achieved a 90.3% and 83.3% accuracy for the XHSLF and ADNI dataset and outperformed fractal connectivity and Pearson correlation by 8% and 17.7% for the XHSLF dataset and 11.63% and 13.3% for the ADNI dataset, respectively. The proposed method also performs better in terms of accuracy, sensitivity, specificity and AUC values when compared to similar published research. The findings of this study indicate the great potential of using the nonfractal connectivity as the biomarker for the diagnosis of AD. However, further investigation needs to be conducted with a larger dataset before it can be used in clinical applications.

## Figures and Tables

**Table 1:** Demographic and neuropsychological information of normal controls (NC) and Alzheimer’s disease (AD) patients related to gender, age, mini-mental state examination (MMSE) and clinical dementia rating (CDR) from Xuanwu Hospital (XH), Santa Lucia Foundation (SLF) and ADNI.

	XH (21) + SLF (10)	XH	ADNI	
	NC	AD	NC	AD
No. of Subject	31	35	30	30
Gender (M/F)	14/17	17/18	13/17	15/15
Age (Year)	63 ± 8.85	65.8 ± 8.3	75.25 ± 6.9	72.81 ± 7.17
MMSE	28.9 ± 1.035	10.1 ± 6.7	24–30	20–26
CDR	0	1–3	0	0.5–1

**Table 2:** Data acquisition protocol for dataset from Xuanwu Hospital (XH), Santa Lucia Foundation (SLF) and ADNI.

Acquisition Protocol			
	**XH**	**SLF**	**ADNI**
Field Strength	3.0 Tesla	3.0 Tesla	3.0 Tesla
Flip Angle	90.0 degrees	70.0 degrees	80.0 degrees
Manufacturer	Siemens	Siemens	Philips
Matrix X	64.0 pixels	64.0 pixels	64.0 pixels
Matrix Y	64.0 pixels	64.0 pixels	64.0 pixels
Slices	5440	7040	6720
Time points	170	220	140
TR	2000.0 ms	2080.0 ms	3000.0 ms
TE	30.0 ms	30.0 ms	30.0 ms
FOV	220 mm × 220 mm	256 mm × 224 mm	212 mm × 212 mm
Slice Thickness	3 mm	2.5 mm	3.3 mm

**Table 3:** Ten-fold cross-validation classification accuracy (in %) of AD vs. NC using k-nearest neighbor (KNN), decision tree, bagged trees and SVM for XHSLF and ADNI dataset.

Classifier	Nonfractal		Fractal		Pearson Correlation	
	XHSLF	ADNI	XHSLF	ADNI	XHSLF	ADNI
KNN (Fine)	80.64 ± 2.51	72.34 ± 3.66	79.96 ± 0.78	58.83 ± 2.69	63.22 ± 1.19	63.32 ± 2.58
Decision tree (Fine)	63.72 ± 4.27	59.51 ± 3.41	60.66± 5.05	54.66 ± 6.33	60.98 ± 4.25	54.02 ± 5.13
Bagged trees (Fine)	71.78 ± 4.33	62.98 ± 0.64	70.97 ± 2.51	62.52 ± 1.32	62.58 ± 2.65	62.82 ± 0.733
SVM (Linear)	**90.3 ± 1.23**	**83.3 ± 0.67**	82.3 ± 0.51	71.67 ± 3.15	72.6 ± 0.71	70 ± 0.76

**Table 4:** Accuracy (in %) of the subspace discriminant ensemble classifier using 820 top-most significant connections for XHSLF dataset and 630 top-most significant connections for ADNI dataset of nonfractal, fractal and Pearson correlation. The value in the bracket is the *p*-value at 820 (XHSLF) or 630 (ADNI) top-most significant connections.

Dataset	Nonfractal	Fractal	Pearson Correlation
XHSLF	90.3 (0.05)	72.6 (0.007)	51.6 (0.009)
ADNI	83.3 (0.05)	63.3 (0.012)	68.3 (0.0006)

**Table 5:** Sensitivity, specificity, accuracy, precision, FPR (in %) and AUC of classification of AD vs. NC based on functional connectivity of rs-fMRI and support vector machine for dataset XHSLF and ADNI.

Evaluation Metric	Nonfractal		Fractal		Pearson Correlation	
	XHSLF	ADNI	XHSLF	ADNI	XHSLF	ADNI
Sensitivity	87.87	100	77.77	70.96	68.42	71.42
Specificity	93.1	75	88.46	72.41	79.16	68.75
Accuracy	90.3	83.3	82.3	71.6	72.6	70
Precision	93.54	66.66	90.3	73.3	83.87	66.66
FPR	6.89	25	11.5	27.5	20.83	31.25
AUC	0.98	0.93	0.88	0.72	0.73	0.70

**Table 6:** 10-fold cross-validation classification accuracy (in %) of AD vs. NC using k-nearest neighbor (KNN), decision tree, bagged trees and SVM for XHSLF+ADNI dataset, which had a total of 122 subjects.

Classifier	Nonfractal	Fractal	Pearson Correlation
	XHSLF+ADNI	XHSLF+ADNI	XHSLF+ADNI
KNN (Fine)	**90.2 ± 0.02**	**73.8 ± 1.85**	60.26 ± 1.13
Decision tree (Fine)	59.8 ± 2.75	58.13 ± 1.17	58.19 ± 2.99
Bagged trees	67.2 ± 0.99	65.93 ± 2.58	64.84 ± 1.88
SVM (linear)	82.8 ± 0.71	66.49 ± 1.42	**73 ± 1.45**

**Table 7:** Sensitivity, specificity, accuracy, precision, FPR (in %) and AUC of classification of AD vs. NC based on functional connectivity of rs-fMRI for XHSLF+ADNI dataset.

Evaluation Metric	Nonfractal	Fractal	Pearson Correlation
	XHSLF+ADNI	XHSLF+ADNI	XHSLF+ADNI
Sensitivity	91.5	75.4	70.5
Specificity	88.88	72.3	75.9
Accuracy	90.2	73.8	73
Precision	88.52	70.49	78.6
FPR	11.11	27.69	24.07
AUC	0.9	0.74	0.77

**Table 8:** Comparison with previous studies in terms of accuracy (%), sensitivity (%), specificity (%) and AUC.

Method	Features	Dataset (Number of Subjects)		Evaluation Metric			
		NC	AD	Accuracy	Sensitivity	Specificity	AUC
De Vos et al. (2018)	Functional connectivity	173	77	79	86	71	0.85
Kasani et al. (2021)	Correlation connectivity	173	74	82.75	82.75	-	-
Zhu et al. (2022)	Functional connectivity	45	44	82.02	-	-	-
**Our Proposed Method (XHSLF)**	Nonfractal connectivity	31	31	90.3	87.87	93.1	0.98
**Our Proposed Method (ADNI)**	Nonfractal connectivity	30	30	83.3	100	75	0.93
**Our Proposed Method (XHSLF+ADNI)**	Nonfractal connectivity	61	61	90.2	91.5	88.88	0.9

## Abbreviations

The following abbreviations are used in this manuscript:

AAL	Automated Anatomical Labeling
AD	Alzheimer’s Disease
ADNI	Alzheimer’s Disease Neuroimaging Initiative
ANOVA	Analysis of Variance
ARIMA	Auto Regressive Fractionally Integrated Moving Average
ARMA	Auto Regressive Moving Average
BOLD	Blood Oxygen Level Dependent
CDR	Clinical Dementia Rating
CONN	Connectivity toolbox
CSF	Cerebrospinal Fluid
DPABI	Data Processing & Analysis for Brain Imaging
DWT	Discrete Wavelet Transform
EEG	Electroencephalography
FGN	Fractional Gaussain Noise
FIN	Fractionally Integrated Noise
FIP	Fractal Integrated Process
fMRI	Functional Magnetic Resonance Imaging
LM	Long Memory
MCI	Mild Cognitive Impairment
ML	Machine Learning
MMSE	Mini Mental State Examination
NC	Normal Controls
PET	Positron Emission Tomography
SLF	Santa Lucia Foundation
sMRI	Structural Magnetic Resonance Imaging
XH	Xuanwu Hospital
XHSLF	Xuanwu Hospital Santa Lucia Foundation
